# Prognostic Nutritional Index and In-Hospital Mortality After Coronary Artery Bypass Grafting: An Exploratory Analysis in Relation to Surgical Risk Scores

**DOI:** 10.3390/nu18122001

**Published:** 2026-06-20

**Authors:** Burak Toprak, Nihat Söylemez, Menaf Akın Sert, Özkan Karaca, Mustafa Ekici, Ali Orçun Sürmeli, Abdulkadir Bilgiç, Samet Yımaz, Sonay Oğuz, Mehmet Ballı, Rıdvan Bora

**Affiliations:** 1 Department of Cardiovascular Surgery, Mersin City Education and Research Hospital, 33240 Mersin, Turkey; sonayoguz1973@gmail.com; 2 Department of Cardiology, Mersin City Education and Research Hospital, 33240 Mersin, Turkey; drnihatsylmz@gmail.com (N.S.); md.ozkrc@gmail.com (Ö.K.); orcun_surmeli@hotmail.com (A.O.S.); dr_mehmetballi@hotmail.com (M.B.); dr.ridvanbora@outlook.com (R.B.); 3 Department of Cardiovascular Surgery, Mersin University Faculty of Medicine Hospital, 33110 Mersin, Turkey; menafsert@gmail.com (M.A.S.); bilgicabdulkadir@gmail.com (A.B.); 4 Department of Emergency Medicine, Mersin Provincial Health Directorate, 33060 Mersin, Turkey; dr.mustafaekici@hotmail.com; 5 Department of Cardiology, Başkent University Adana Research Center, 01250 Adana, Turkey; sametyilmaz.dr@gmail.com

**Keywords:** coronary artery bypass grafting, in-hospital mortality, prognostic nutritional index, nutritional status, immune status, surgical risk assessment

## Abstract

**Background:** Coronary anatomical complexity is commonly used for perioperative risk assessment in patients undergoing coronary artery bypass grafting (CABG), although it may not fully reflect systemic biological vulnerability. This study aimed to evaluate the association between the Prognostic Nutritional Index (PNI), a nutritional–immune marker derived from serum albumin and lymphocyte counts, and in-hospital mortality after CABG in relation to coronary anatomical complexity and established surgical risk scores. **Methods:** In this single-center retrospective cohort study, 324 consecutive patients who underwent isolated CABG between April 2024 and April 2025 were analyzed. The PNI was calculated according to the standard Onodera formula using preoperative serum albumin and total lymphocyte count. Associations with in-hospital mortality were evaluated using univariable and multivariable logistic regression analyses. Discriminative performance was assessed using receiver operating characteristic curve analysis, while exploratory analyses evaluating the additional prognostic contribution of the PNI beyond surgical risk scores were performed using nested model comparison and reclassification analyses. Internal validation and calibration analyses were also performed. **Results:** In-hospital mortality occurred in 26 patients. Preoperative and postoperative PNI values were significantly lower in patients who experienced in-hospital mortality. In multivariable analysis, the postoperative PNI remained independently associated with in-hospital mortality, whereas the preoperative PNI lost statistical significance after adjustment for clinical, renal, and surgical risk parameters. Receiver operating characteristic analysis demonstrated modest discriminative ability for the preoperative PNI (AUC: 0.742, 95% CI: 0.661–0.823). Exploratory analyses suggested a modest improvement in model discrimination and risk classification after the addition of the PNI to STS-based models; however, the overall incremental prognostic contribution remained limited. Calibration and internal validation analyses demonstrated acceptable agreement between predicted and observed mortality risk. **Conclusions:** The postoperative PNI demonstrated a stronger and independent association with in-hospital mortality than the preoperative PNI, suggesting that early postoperative nutritional–immune deterioration may reflect the magnitude of perioperative physiological stress and evolving clinical deterioration after CABG. Although lower preoperative PNI values were associated with mortality in univariable analyses, this association was no longer statistically significant after adjustment for clinical, renal, and surgical risk parameters. These findings indicate that postoperative nutritional–immune status may provide complementary biological information beyond conventional risk models; however, its clinical utility requires confirmation in larger prospective multicenter studies.

## 1. Introduction

Coronary artery bypass grafting (CABG) is a well-established surgical treatment with proven efficacy in controlling symptoms and improving long-term survival in patients with advanced coronary artery disease [1]. Despite substantial advances in surgical techniques, perioperative care, myocardial protection strategies, and intensive care management, in-hospital mortality after CABG remains a clinically significant problem associated with prolonged hospitalization, organ dysfunction, infectious complications, and increased healthcare burden [2]. Therefore, identifying reliable markers capable of predicting perioperative mortality risk in both the preoperative and early postoperative periods remains critically important for patient selection, perioperative optimization, and individualized risk stratification.

In contemporary clinical practice, perioperative risk assessment in CABG patients is based on a combination of anatomical, clinical, and procedural parameters. The SYNTAX score is one of the most widely used tools for evaluating the anatomical complexity of coronary artery disease and plays an important role in determining revascularization strategies [3]. Higher SYNTAX scores have been associated with increased procedural complexity, prolonged cardiopulmonary bypass duration, and a greater incidence of perioperative adverse events [4]. However, anatomical complexity alone does not fully explain postoperative outcomes, as patients with similar coronary anatomical burden may demonstrate markedly different perioperative clinical courses and mortality rates [5].

For this reason, multivariable surgical risk models such as EuroSCORE II and the Society of Thoracic Surgeons (STS) score are widely used in routine clinical practice to estimate perioperative mortality risk by integrating patient age, comorbidities, ventricular function, and operative characteristics [6]. Although these models have been validated in large populations and remain central components of modern surgical risk assessment, they may reflect nutritional reserve, immune competence, inflammatory activation, and biological frailty only indirectly at the individual patient level [7]. Importantly, conventional risk models do not directly quantify nutritional–immune status, despite increasing evidence suggesting that perioperative systemic vulnerability substantially contributes to postoperative complications and mortality. This limitation highlights the need to investigate complementary biomarkers capable of reflecting patient-specific biological reserve beyond traditional surgical risk scores.

In recent years, increasing attention has focused on the interaction between nutritional status, systemic inflammation, and postoperative outcomes in cardiovascular surgery. Malnutrition is associated with hypoalbuminemia, lymphocyte dysfunction, impaired cellular immunity, delayed tissue healing, and increased susceptibility to infection and organ failure [8]. Within this pathophysiological framework, the Prognostic Nutritional Index (PNI), calculated using serum albumin levels and peripheral lymphocyte counts, has emerged as a practical biomarker reflecting both nutritional and immune reserve [9,10,11]. Previous studies have demonstrated associations between low PNI values and adverse outcomes in oncologic surgery, critically ill patients, and various cardiovascular disease populations [10]. More recently, several investigations have also suggested that a reduced preoperative PNI may be associated with increased mortality and postoperative morbidity after CABG [1,12].

Beyond mortality, impaired nutritional–immune reserve may contribute to prolonged mechanical ventilation, extended intensive care unit stay, postoperative infection, sepsis, and delayed recovery after cardiac surgery [13,14,15,16]. Because cardiopulmonary bypass induces a substantial systemic inflammatory response, biomarkers integrating nutritional and immune status may provide complementary prognostic information beyond conventional anatomical and surgical risk models [14,15,16].

Although the number of studies evaluating the PNI in CABG patients has increased in recent years, most available studies have investigated the PNI in isolation, with limited integration into established surgical risk assessment strategies. In particular, it remains unclear whether nutritional–immune status may provide additional prognostic value beyond conventional surgical risk scores and coronary anatomical complexity in predicting in-hospital mortality after CABG. Clarifying this relationship may contribute to a more comprehensive and biologically integrated perioperative risk stratification approach.

Therefore, the aim of the present study was to evaluate the association between the Prognostic Nutritional Index and in-hospital mortality after CABG and to investigate whether the PNI may provide incremental prognostic value beyond established surgical risk models, including STS score, EuroSCORE II, and SYNTAX score. In addition, we aimed to explore the relationship between nutritional–immune status, perioperative adverse clinical events, and postoperative mortality in patients undergoing CABG.

## 2. Materials and Methods

### 2.1. Data Collection

#### 2.1.1. Study Design

This study was designed as a single-center, retrospective, observational cohort study. The study population consisted of consecutive patients who underwent isolated coronary artery bypass grafting at the Department of Cardiovascular Surgery, Mersin University Faculty of Medicine, between 1 April 2024 and 1 April 2025. All surgical procedures were performed using conventional on-pump CABG techniques under cardiopulmonary bypass. No off-pump CABG procedures were included in the study cohort. Owing to the retrospective nature of the study, no interventions were made regarding patient management, surgical techniques, or perioperative treatment strategies, and all clinical decisions were made in accordance with routine clinical practice.

The study was conducted in compliance with the principles of the Declaration of Helsinki and was approved by the Local Ethics Committee for Clinical Research of Mersin University (approval date: 30 April 2025; decision number: 2025/455). Written informed consent was routinely obtained from all patients before surgical and hospitalization procedures as part of standard clinical practice, including consent regarding the use of anonymized clinical data for scientific purposes. Due to the retrospective design and the analysis of anonymized data, the requirement for additional study-specific informed consent was waived by the ethics committee.

#### 2.1.2. Data Collection

Patients who underwent isolated CABG within the specified time frame and had complete clinical, laboratory, and angiographic data were included in the study. A patient flow diagram detailing the numbers of screened, excluded, and finally included patients was constructed and reported in accordance with the STROBE recommendations for observational studies. Patients undergoing concomitant valve or aortic surgery, those with hemodynamic instability requiring emergency surgery, active infection, malignancy, a history of chronic inflammatory disease, use of immunosuppressive therapy, or incomplete clinical or laboratory data required for PNI calculation or outcome assessment were excluded. Among the initially screened patients, 27 were excluded because of incomplete clinical or laboratory data required for PNI calculation or outcome assessment. The reasons for exclusion and the final study selection process were summarized in the STROBE-compatible patient flow diagram. Because of the retrospective study design and the relatively limited proportion of missing data, complete-case analysis was performed and no imputation method was applied. Nevertheless, the possibility that missing data were not completely random cannot be fully excluded. Patients presenting with cardiogenic shock, ongoing cardiopulmonary resuscitation, mechanical circulatory support requirement, or severe hemodynamic instability requiring emergent surgery were excluded, and the analyzed cohort therefore predominantly consisted of clinically stable elective or urgent-but-stabilized CABG patients.

Demographic characteristics, clinical comorbidities, perioperative surgical variables, intensive care unit data, and postoperative adverse clinical events were retrospectively obtained from the hospital information management system. Evaluated perioperative adverse clinical events included prolonged mechanical ventilation, prolonged intensive care unit stay, prolonged inotropic support requirement, postoperative pneumonia, and sepsis. Prolonged mechanical ventilation was defined as the requirement for invasive mechanical ventilatory support for more than 24 h after surgery. Prolonged intensive care unit stay was defined as ICU hospitalization exceeding 72 h postoperatively. Prolonged inotropic support was defined as the continued requirement for inotropic or vasopressor support beyond the first 24 postoperative hours. Postoperative pneumonia was diagnosed according to clinical, radiological, and microbiological findings documented by the treating physicians, whereas sepsis was defined according to contemporary clinical diagnostic criteria based on systemic infection-associated organ dysfunction.

In-hospital mortality was evaluated as all-cause mortality occurring during the index hospitalization following CABG surgery, regardless of postoperative duration. Mortality data were obtained from hospital electronic medical records and the national health registry system. Coronary anatomical complexity was assessed using the SYNTAX score based on preoperative coronary angiography, and patients were categorized into low, intermediate, and high SYNTAX groups according to standard classification criteria. In addition, perioperative surgical risk assessment was performed using both EuroSCORE II and the Society of Thoracic Surgeons (STS) risk score according to standard validated calculation models.

Nutritional and immune status was evaluated using the Prognostic Nutritional Index (PNI), calculated from preoperative laboratory data. Preoperative laboratory parameters, including serum albumin and total lymphocyte count, were measured as part of routine clinical evaluation using standardized automated laboratory systems in the hospital central laboratory. Preoperative laboratory measurements, including serum albumin and total lymphocyte count, were obtained within 24 h prior to surgery as part of routine preoperative assessment. This timing was standardized across all patients according to institutional protocols, ensuring consistency in the evaluation of baseline nutritional and immune status. Such an approach is consistent with previous cardiac surgery studies, in which preoperative biomarkers are typically measured within the immediate preoperative period to reflect the patient’s true physiological reserve before surgical stress. Serum albumin levels were determined by a bromocresol green colorimetric method, while lymphocyte counts were obtained from complete blood count analysis using an automated hematology analyzer. All measurements were performed according to the manufacturer’s instructions and internal quality control procedures, ensuring analytical consistency and reproducibility.

The PNI was calculated according to the original Onodera formula using serum albumin expressed in g/dL and total lymphocyte count expressed as cells/mm^3^ [11]:

**PNI = 10 × albumin (g/dL) + 0.005 × total lymphocyte count (cells/mm^3^)**

Because serum albumin values in the laboratory records were reported in g/L, albumin values were converted to g/dL by dividing by 10. Similarly, lymphocyte counts reported as ×10^9^/L were converted to cells/mm^3^ by multiplying by 1000. Accordingly, the formula was equivalent to albumin (g/L) + 5 × lymphocyte count (×10^9^/L). All PNI values, ROC analyses, cut-off values, and subgroup classifications were recalculated using this standard Onodera PNI scale. The PNI was analyzed both as a continuous variable and as a categorical variable according to the optimal cut-off value determined by ROC analysis. The preoperative PNI was evaluated as a marker of baseline nutritional–immune reserve for perioperative risk assessment, whereas the postoperative PNI was analyzed separately as an exploratory early postoperative prognostic marker reflecting the physiological and inflammatory response to surgical stress. Postoperative laboratory measurements used for postoperative PNI calculation were obtained within the first 24 h after surgery according to routine institutional intensive care unit protocols.

### 2.2. Data Analysis

#### 2.2.1. Statistical Analysis

Statistical analyses were performed according to a predefined analysis plan to identify variables associated with in-hospital mortality. In this study, in-hospital mortality was defined as all-cause mortality occurring during the index hospitalization following coronary artery bypass grafting, regardless of postoperative duration. Throughout the manuscript, the term “in-hospital mortality” was consistently used to define the primary study endpoint. The distribution of continuous variables was assessed using the Kolmogorov–Smirnov and Shapiro–Wilk tests. Normally distributed variables were expressed as mean ± standard deviation, whereas non-normally distributed variables were presented as median (interquartile range). Variables demonstrating substantial skewness were additionally reviewed using distribution histograms and normality testing results before selection of the final descriptive format.

For group comparisons, Student’s *t*-test or the Mann–Whitney U test was used for continuous variables, depending on data distribution, while the chi-square test or Fisher’s exact test, as appropriate, was applied for categorical variables.

To identify potential predictors of in-hospital mortality, univariable logistic regression analyses were initially performed. Variables demonstrating statistical significance in univariable analyses and variables considered clinically relevant based on perioperative cardiovascular risk assessment principles were subsequently evaluated in multivariable logistic regression models. Established surgical risk scores, including EuroSCORE II and the Society of Thoracic Surgeons score, were incorporated into the statistical analyses to evaluate the incremental prognostic contribution of Prognostic Nutritional Index beyond conventional perioperative risk models. In addition, clinically relevant perioperative variables such as pulmonary arterial hypertension, ejection fraction, graft number, postoperative adverse clinical events, and renal function parameters were evaluated according to their biological plausibility and univariable associations.

Variables with a *p* value < 0.10 in univariable analyses were subsequently included in multivariable logistic regression models. A threshold of *p* < 0.10 was selected at the univariable screening stage to avoid the premature exclusion of potentially relevant predictors and to allow clinically meaningful variables with borderline statistical significance to be considered during multivariable model construction. In addition, age, STS score, and SYNTAX score were retained in the multivariable models irrespective of univariable statistical significance because of their established clinical relevance in perioperative cardiovascular risk assessment. Therefore, the final exploratory parsimonious models included both statistically selected variables and clinically forced covariates.

To minimize the risk of overfitting, particular attention was paid to the ratio between the number of outcome events and the number of variables included in the multivariable models. In the present study, 26 mortality events were observed, and the number of covariates included in each multivariable model was restricted to ensure an adequate events-per-variable (EPV) ratio. Consistent with established methodological recommendations suggesting a minimum of 10 events per variable, the final models were constructed using a limited number of clinically relevant predictors selected based on univariable screening and biological plausibility. This approach was adopted to improve model stability and to reduce the risk of overfitting.

Intraoperative variables such as cardiopulmonary bypass duration and cross-clamp time were not included in the primary preoperative multivariable models because these parameters occur after the initial preoperative risk assessment phase and may function as mediators rather than true baseline confounders. Nevertheless, supplementary sensitivity analyses including cross-clamp duration and cardiopulmonary bypass time demonstrated similar overall findings, with the postoperative PNI remaining associated with in-hospital mortality after adjustment for operative complexity parameters. Adjusting for such intraoperative variables could introduce overadjustment bias and potentially obscure the independent prognostic value of preoperative biological markers. Therefore, the primary multivariable analyses intentionally focused on clinically applicable perioperative risk prediction models. During model construction, the potential presence of multicollinearity among variables was assessed using variance inflation factor (VIF) analysis, and variables demonstrating significant multicollinearity were excluded from the final models.

The discriminatory ability of the PNI for in-hospital mortality was evaluated using Receiver Operating Characteristic (ROC) curve analysis, and the area under the curve (AUC) was reported with a 95% confidence interval. The optimal PNI cut-off value was determined based on the Youden index. Because the ROC-derived cut-off value was both generated and evaluated within the same study cohort, the resulting threshold should be interpreted as exploratory and hypothesis-generating rather than externally validated.

To further evaluate the incremental prognostic contribution of the PNI beyond established surgical risk models, nested multivariable models including STS score alone and combined STS + PNI models were constructed. Differences in discriminative performance between models were evaluated using ROC curve comparison analyses. Exploratory model reclassification performance was additionally assessed using continuous net reclassification improvement (NRI) and integrated discrimination improvement (IDI). Internal validation was performed using bootstrap resampling with 1000 iterations to estimate optimism-corrected discrimination and calibration metrics. Model calibration was evaluated using calibration slope, calibration intercept, Brier score, the Hosmer–Lemeshow goodness-of-fit test, and visual inspection of calibration plots.

All statistical tests were two-sided, and a *p* value < 0.05 was considered statistically significant. An a priori sample size estimation was performed using G*Power software (version 3.1, Heinrich Heine University, Düsseldorf, Germany). Based on a conservative assumption of a moderate effect size (Cohen’s d = 0.50) for the difference in the preoperative Prognostic Nutritional Index between patients with and without in-hospital mortality, a two-tailed independent-samples *t*-test with an alpha level of 0.05 and a target power of 0.90 was planned. Using the observed group allocation ratio (Alive/Mortality ≈ 5.6:1), the required minimum sample size was estimated to be approximately 330 patients. Given that the present study included 324 patients, the achieved sample size was considered sufficient for detecting clinically meaningful differences with adequate statistical power.

#### 2.2.2. Software

All statistical analyses were performed using Python (version 3.12). Data processing and cleaning were conducted using the pandas and numpy libraries, statistical analyses were performed with scipy and statsmodels, and ROC analysis and classification performance assessments were carried out using the scikit-learn package. Bootstrap validation procedures and calibration analyses were also performed within the Python statistical environment. The analysis workflow was conducted in accordance with principles of reproducibility.

## 3. Results

As shown in Table 1, patients who experienced in-hospital mortality had a substantially higher perioperative risk profile than survivors. They were older and more frequently had pulmonary arterial hypertension, lower ejection fraction, higher SYNTAX scores, and higher surgical risk scores as assessed by STS and EuroSCORE II (all *p* < 0.01). Mortality was also associated with a greater graft number and a markedly increased incidence of adverse postoperative events, including prolonged mechanical ventilation, prolonged intensive care unit stay, prolonged inotropic support, pneumonia, and sepsis (all *p* ≤ 0.031) (Table 1).

**Figure 1:**
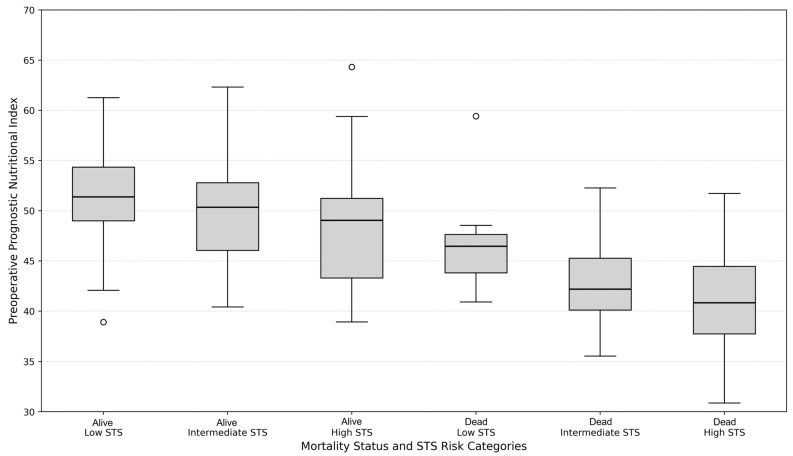
Distribution of preoperative Prognostic Nutritional Index values according to in-hospital mortality status and STS risk categories. Box plots display the median, interquartile range, and overall distribution of preoperative Prognostic Nutritional Index values in patients with and without in-hospital mortality (Alive and Mortality), stratified into low, intermediate, and high STS risk categories. Across all STS risk categories, patients with in-hospital mortality generally demonstrated lower PNI distributions compared with survivors, particularly within low- and intermediate-risk groups.

The distribution of preoperative Prognostic Nutritional Index differed between patients with and without in-hospital mortality across all STS risk categories. In the low STS risk category, median PNI values were lower in patients who experienced in-hospital mortality compared with those without mortality. A similar pattern was observed in the intermediate STS risk group, where patients in the Mortality group demonstrated lower PNI values relative to the Non-Mortality group. In the high STS risk category, preoperative PNI values also remained generally lower in patients with in-hospital mortality compared with survivors, although partial overlap between groups was observed. Overall, across low, intermediate, and high STS risk categories, patients who experienced in-hospital mortality consistently exhibited lower preoperative PNI distributions than those without mortality (Figure 1).

Correlation analysis demonstrated a weak but statistically significant negative association between preoperative Prognostic Nutritional Index and SYNTAX score (Spearman ρ = −0.124, *p* = 0.025). In addition, preoperative Prognostic Nutritional Index showed moderate negative correlations with both STS score (Spearman ρ = −0.418, *p* < 0.001) and EuroSCORE II (Spearman ρ = −0.391, *p* < 0.001) (Table 2). These findings suggest that lower nutritional–immune reserve was associated not only with greater coronary anatomical complexity but also with higher overall predicted surgical risk (Table 2).

Univariable logistic regression analysis identified several demographic, clinical, laboratory, and postoperative variables associated with in-hospital mortality (Table 3). Older age, pulmonary arterial hypertension, lower ejection fraction, higher SYNTAX score, higher STS score, higher EuroSCORE II, and greater graft number were all associated with increased mortality risk. The preoperative PNI was significantly associated with mortality; however, the association was more pronounced for the postoperative PNI. Similarly, lower albumin levels, impaired renal function, and adverse postoperative clinical events were associated with an increased risk of in-hospital mortality. Among postoperative variables, prolonged mechanical ventilation, prolonged intensive care unit stay, prolonged inotropic support, pneumonia, and sepsis demonstrated particularly strong associations with mortality (Table 3).

**Figure 2:**
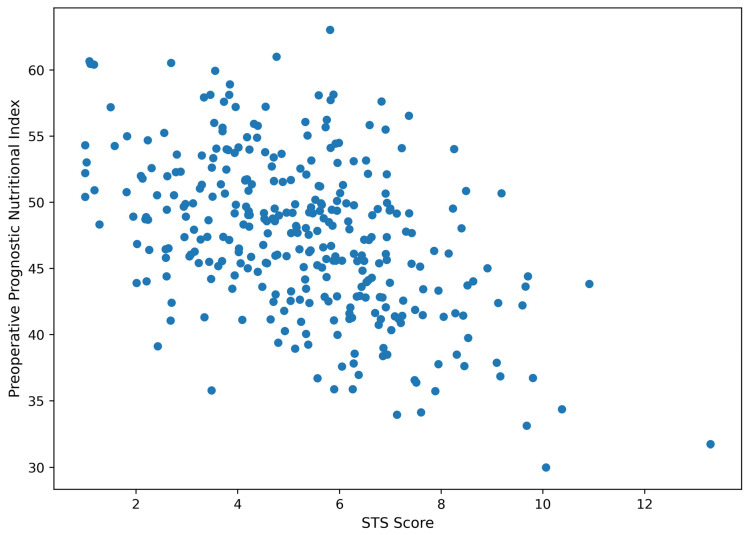
Scatter plot demonstrating the relationship between preoperative Prognostic Nutritional Index and STS score. Each point represents an individual patient. A weak-to-moderate inverse relationship was observed between preoperative PNI and STS score, indicating that lower nutritional–immune reserve tended to be associated with higher predicted surgical risk. However, substantial overlap between patients remained evident across the full spectrum of STS scores, suggesting that nutritional–immune status and conventional surgical risk assessment may represent partially distinct clinical domains.

The relationship between preoperative Prognostic Nutritional Index and STS score was further evaluated using scatter plot visualization (Figure 2). The distribution of data points demonstrated a generally inverse trend between the two variables, supporting the results of the correlation analysis. Lower preoperative PNI values were more frequently observed in patients with higher STS scores, whereas higher PNI values tended to cluster within lower surgical risk ranges. Nevertheless, considerable overlap between patients remained across the spectrum of STS scores, indicating that nutritional–immune status may provide complementary biological information beyond traditional surgical risk assessment tools.

In the exploratory parsimonious multivariable models, the association between the preoperative PNI and in-hospital mortality was attenuated after adjustment for demographic characteristics, renal function, surgical risk scores, and coronary anatomical complexity. The preoperative PNI was no longer independently associated with mortality, suggesting that its univariable association may primarily reflect greater baseline clinical vulnerability rather than an independent prognostic effect. In contrast, the postoperative PNI remained independently associated with in-hospital mortality after multivariable adjustment, representing the principal prognostic finding of the study (Table 4).

Among established surgical risk parameters, STS score remained independently associated with mortality in both multivariable models, whereas EuroSCORE II lost statistical significance after adjustment. Renal dysfunction also remained an independent predictor of mortality, with both urea and creatinine demonstrating significant associations in the preoperative and postoperative models.

In addition, prolonged mechanical ventilation and prolonged intensive care unit stay remained independently associated with increased mortality risk in the postoperative model (Table 4).

In patients with low STS risk, in-hospital mortality rates were significantly higher in the low PNI group compared with the high PNI group (17.8% vs. 2.0%, *p* = 0.002). Similarly, among patients with intermediate STS risk, the low PNI group demonstrated significantly increased mortality rates compared with patients with high PNI values (16.4% vs. 6.0%, *p* = 0.044). In the high STS risk category, mortality rates remained numerically higher in patients with a low PNI compared with those with a high PNI (32.1% vs. 25.6%); however, this difference did not reach statistical significance (*p* = 0.581) (Table 5).

**Figure 3:**
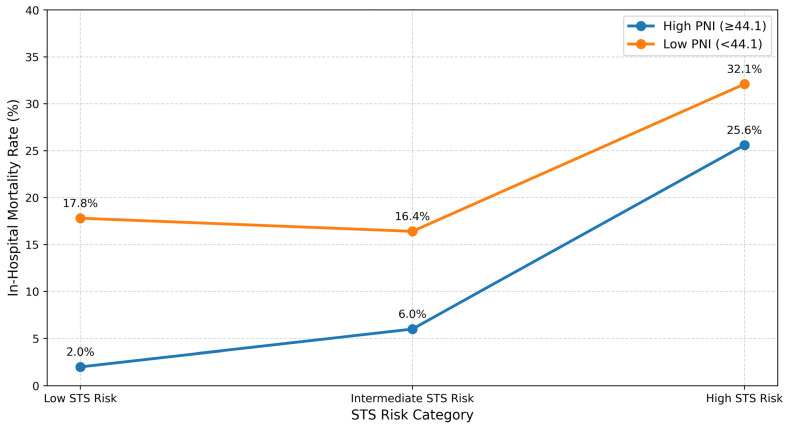
Interaction between preoperative Prognostic Nutritional Index and STS risk categories in predicting in-hospital mortality after coronary artery bypass surgery. In-hospital mortality rates are presented for patients with high and low preoperative PNI values across low, intermediate, and high STS risk categories. Patients with a low PNI consistently demonstrated higher in-hospital mortality rates compared with those with a high PNI across all STS risk groups.

In-hospital mortality rates differed according to combined Prognostic Nutritional Index status and STS risk categories. In patients with low STS risk, in-hospital mortality rates were substantially lower in the high PNI group compared with the low PNI group. A similar pattern was observed in the intermediate STS risk category, where patients with a low PNI demonstrated higher mortality rates than those with a high PNI. In the high STS risk category, mortality rates increased in both groups, with consistently higher mortality observed among patients with a low PNI compared with those with a high PNI across all levels of surgical risk (Figure 3).

Receiver operating characteristic curve analysis demonstrated that preoperative Prognostic Nutritional Index had statistically significant discriminative ability for predicting in-hospital mortality after coronary artery bypass surgery. The area under the curve was 0.742 (95% CI: 0.661–0.823, *p* < 0.001), indicating acceptable predictive performance. The ROC-derived PNI cut-off value for in-hospital mortality was identified as 44.1, yielding a sensitivity of 73.1% and a specificity of 68.8%, with a Youden index of 0.419 (Table 6). These findings suggest that reduced preoperative nutritional–immune reserve was associated with increased postoperative mortality risk during hospitalization. However, because this cut-off value was both derived and evaluated within the same cohort, it should still be interpreted as exploratory and hypothesis-generating rather than as a clinically validated decision threshold (Table 6).

**Figure 4:**
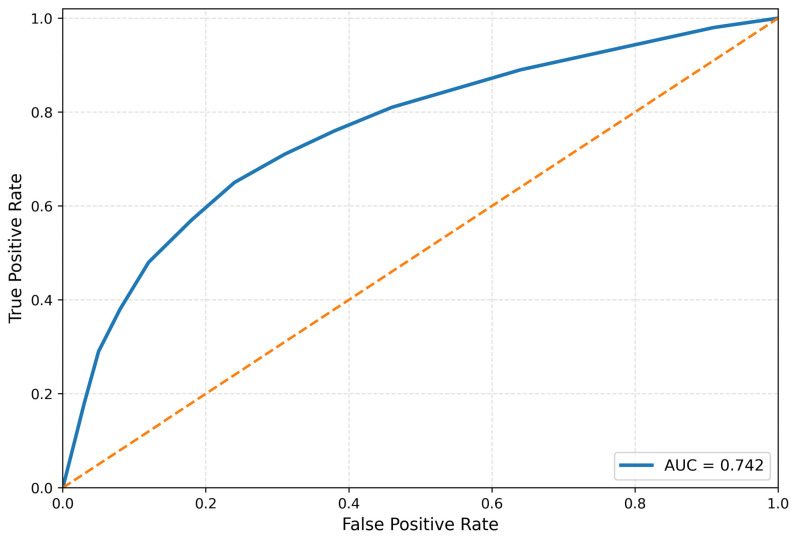
Receiver operating characteristic (ROC) curve of preoperative Prognostic Nutritional Index for predicting in-hospital mortality after coronary artery bypass surgery. The curve illustrates the relationship between sensitivity and specificity across a range of preoperative PNI thresholds, with the diagonal reference line indicating no discriminative ability. The area under the curve was 0.742, indicating acceptable discriminative performance for predicting in-hospital mortality.

Receiver operating characteristic analysis demonstrated that preoperative Prognostic Nutritional Index had statistically significant discriminative ability for predicting in-hospital mortality after coronary artery bypass surgery. The area under the curve was 0.742, indicating acceptable predictive performance (Figure 4).

To further explore the incremental prognostic contribution of preoperative PNI beyond established surgical risk assessment, two exploratory nested models were constructed. The addition of the preoperative PNI to the STS-based model resulted in improved discrimination for in-hospital mortality, with the area under the curve increasing from 0.801 to 0.847. Exploratory reclassification analyses also suggested improved risk stratification following the inclusion of the PNI.

Internal bootstrap validation demonstrated limited optimism, with an optimism-corrected AUC of 0.829 for the combined model. Calibration analyses showed acceptable agreement between predicted and observed mortality risk, and the Hosmer–Lemeshow test demonstrated satisfactory calibration for both models (Table 7). These findings suggest that the preoperative PNI may provide complementary prognostic information beyond conventional surgical risk assessment; however, the incremental benefit should be interpreted cautiously given the retrospective design and limited number of outcome events (Table 7).

**Figure 5:**
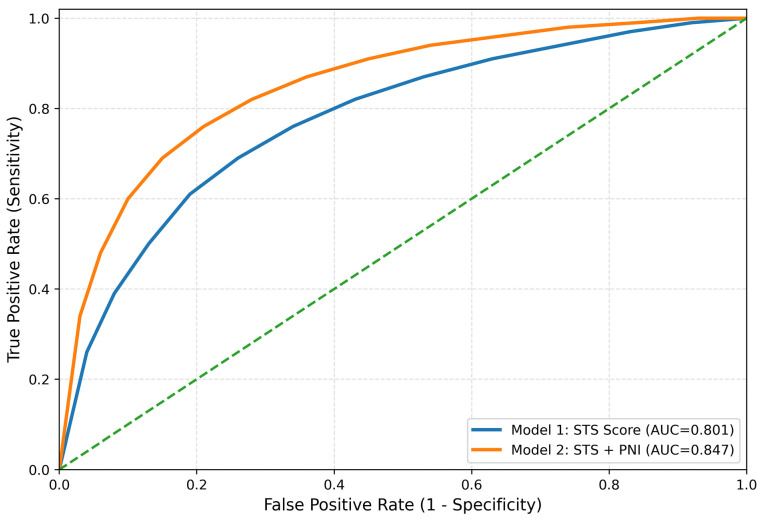
Receiver operating characteristic curve comparison of STS score alone and the combined STS score plus Prognostic Nutritional Index model for prediction of in-hospital mortality after coronary artery bypass surgery. The combined model demonstrated improved discriminative performance compared with the STS-only model. The area under the curve increased from 0.801 for the STS-only model to 0.847 for the combined STS + PNI model, suggesting that the addition of nutritional–immune status provided incremental prognostic value beyond conventional surgical risk assessment. The green dashed line indicating that it represents the reference line for no discriminative ability (AUC = 0.50).

Receiver operating characteristic curve comparison demonstrated improved discriminative performance after the addition of preoperative Prognostic Nutritional Index to the STS score for predicting in-hospital mortality after coronary artery bypass surgery. The area under the curve increased from 0.801 for the STS-only model to 0.847 for the combined STS + PNI model, indicating incremental prognostic improvement with the incorporation of nutritional–immune status into conventional surgical risk assessment (Figure 5).

**Figure 6:**
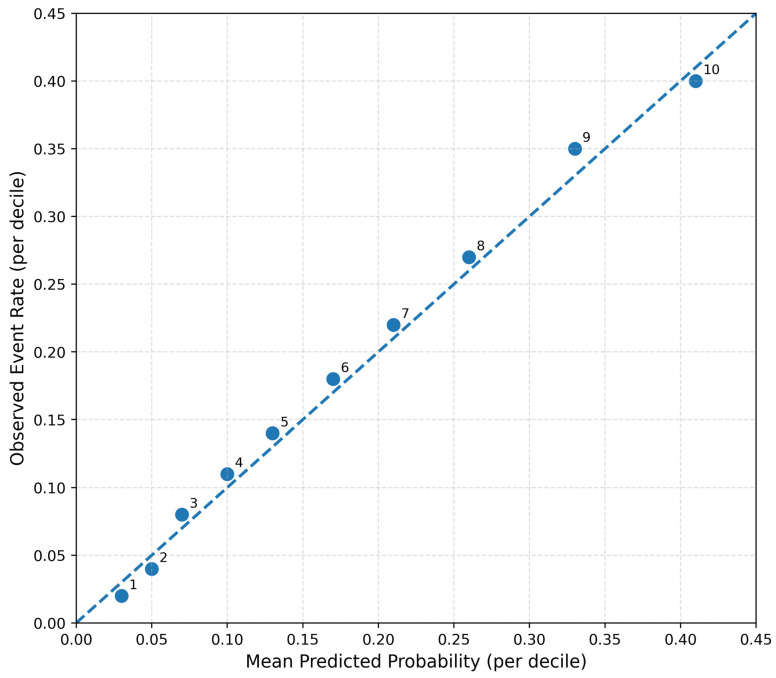
Calibration plot of the combined STS score and Prognostic Nutritional Index model for prediction of in-hospital mortality after coronary artery bypass surgery. The calibration plot demonstrates the agreement between predicted and observed in-hospital mortality probabilities across deciles of predicted risk. Each point represents the mean predicted probability and the corresponding observed event rate within each decile. Although mild deviations from the reference line were observed in several deciles, the overall distribution of calibration points suggested acceptable agreement between predicted and observed mortality risk.

Calibration analysis demonstrated acceptable agreement between predicted and observed in-hospital mortality probabilities for the combined STS + PNI model (Figure 6). Although minor deviations from the reference line were observed in several deciles, the overall distribution of calibration points suggested reasonable model calibration across different levels of predicted risk. However, this calibration assessment reflects internal model performance only and does not establish external generalizability.

## 4. Discussion

In this study, the Prognostic Nutritional Index, reflecting nutritional and immune status, was evaluated in relation to in-hospital mortality in patients undergoing isolated coronary artery bypass grafting. The principal finding of this study was that low preoperative PNI values were associated with increased in-hospital mortality in univariable analyses and appeared to reflect increased systemic biological vulnerability in CABG patients. However, after adjustment for demographic characteristics, renal function parameters, coronary anatomical complexity, and established surgical risk scores, the independent association of the preoperative PNI with mortality was attenuated and no longer statistically significant. In contrast, the postoperative PNI remained independently associated with in-hospital mortality, suggesting that postoperative nutritional–immune deterioration may primarily reflect the magnitude of perioperative physiological stress and evolving postoperative clinical deterioration rather than baseline biological reserve alone. Therefore, the preoperative PNI should not be interpreted as a definitive standalone prognostic marker, although it may provide complementary biological information in perioperative risk assessment.

The PNI is a composite biomarker integrating serum albumin levels and lymphocyte counts and therefore reflects both nutritional reserve and immune competence [10,11]. Serum albumin represents a common endpoint of multiple pathophysiological processes, including protein reserve, inflammatory burden, endothelial dysfunction, capillary leakage, and metabolic stress response [12]. Hypoalbuminemia has been associated with impaired wound healing, increased inflammatory activation, prolonged intensive care requirement, and postoperative organ dysfunction after cardiac surgery [8,12]. Lymphocyte count, on the other hand, is a fundamental indicator of cellular immune competence, and lymphopenia has been linked to increased susceptibility to infection, sepsis, and multiple organ failure [13]. Accordingly, reduced PNI values may reflect impaired systemic biological resilience characterized by diminished tolerance to perioperative inflammatory and metabolic stress.

The present study further demonstrated that patients with in-hospital mortality had significantly higher rates of prolonged mechanical ventilation, prolonged intensive care unit stay, prolonged inotropic support requirement, pneumonia, and sepsis. These findings strongly support the concept that impaired nutritional–immune reserve contributes not only to mortality itself but also to postoperative clinical deterioration and reduced recovery capacity. Cardiopulmonary bypass is known to trigger a substantial systemic inflammatory response characterized by cytokine release, endothelial dysfunction, oxidative stress, immune dysregulation, and metabolic imbalance [14]. Patients with impaired baseline biological reserve may therefore be less capable of tolerating this inflammatory burden, resulting in increased susceptibility to respiratory failure, infection, prolonged organ support, and adverse postoperative outcomes.

In current clinical practice, perioperative cardiac surgical risk assessment primarily relies on validated multivariable models such as EuroSCORE II and the Society of Thoracic Surgeons score [6,7]. Unlike the earlier analytical framework of the present study, both STS and EuroSCORE II were incorporated into the updated analyses. Importantly, STS score remained independently associated with in-hospital mortality in multivariable models, confirming the expected prognostic relevance of established surgical risk assessment systems within the present cohort. However, the addition of the PNI to STS-based models resulted in additional improvement in model discrimination and calibration performance. Specifically, the area under the curve increased from 0.801 for the STS-only model to 0.847 for the combined STS + PNI model, while calibration metrics and exploratory reclassification analyses also demonstrated numerical improvement. These findings suggest that nutritional–immune status may reflect additional dimensions of perioperative biological vulnerability not fully captured by traditional surgical risk models alone.

The relationship between the PNI and mortality should therefore be interpreted within the broader concept of perioperative biological reserve rather than as an isolated nutritional marker alone. Traditional surgical risk scores incorporate demographic and clinical variables but do not directly quantify systemic inflammatory activation, immune competence, frailty burden, or metabolic reserve at the individual patient level [7]. In this context, biomarkers such as the PNI may provide complementary biological information reflecting the patient’s capacity to tolerate perioperative physiological stress. Importantly, however, the incremental prognostic contribution observed after incorporation of the PNI remained modest and exploratory and should therefore be interpreted cautiously within the context of a retrospective single-center cohort.

Coronary anatomical complexity, represented by the SYNTAX score, also demonstrated an association with mortality in univariable analyses. However, anatomical disease burden alone may not fully explain postoperative outcomes after cardiac surgery [3,4,5]. Patients with similar anatomical complexity frequently demonstrate markedly different postoperative clinical trajectories depending on systemic inflammatory burden, nutritional reserve, renal dysfunction, pulmonary hypertension, and perioperative immune competence. In the present study, patients with mortality demonstrated significantly higher STS and EuroSCORE II values in addition to lower PNI levels, suggesting that perioperative outcomes are influenced by both operative risk burden and systemic biological vulnerability. Furthermore, the observation that patients with low PNI values demonstrated increased mortality even within lower STS risk categories supports the concept that nutritional–immune impairment may contribute additional prognostic context beyond traditional surgical risk assessment alone.

One of the most important findings of the present study was the stronger prognostic association observed for the postoperative PNI compared with the preoperative PNI. Unlike the preoperative PNI, which primarily reflects baseline biological reserve before surgical intervention, the postoperative PNI is strongly influenced by perioperative inflammatory activation, surgical trauma, hemodilution, renal dysfunction, fluid shifts, perioperative bleeding, infection, and evolving postoperative organ dysfunction. Therefore, the postoperative PNI likely reflects the severity of the physiological response to surgery rather than baseline patient vulnerability alone. This pathophysiological framework may explain why the postoperative PNI remained independently associated with in-hospital mortality even after multivariable adjustment. Accordingly, the postoperative PNI may be more appropriately interpreted as an exploratory early postoperative monitoring marker reflecting evolving clinical deterioration rather than as a purely preoperative risk stratification tool. This distinction is clinically important when interpreting the prognostic role of the postoperative PNI. Because postoperative measurements were obtained within the first 24 h after surgery, the postoperative PNI may represent an early manifestation of the physiological consequences of surgical stress rather than a purely antecedent risk factor. Therefore, the observed association with mortality should not necessarily be interpreted as evidence of causal prognostic superiority over the preoperative PNI. Instead, the postoperative PNI may function as an integrated marker reflecting the combined effects of baseline biological reserve, perioperative inflammatory activation, and the early development of postoperative complications.

The ROC analyses performed in the present study demonstrated acceptable discriminative performance for the preoperative PNI in predicting in-hospital mortality, with an AUC of 0.742. In addition, incorporation of the PNI into STS-based predictive models resulted in modest numerical improvement in discrimination and exploratory reclassification analyses. Continuous net reclassification improvement and integrated discrimination improvement analyses also demonstrated numerical enhancement following the addition of PNI to STS-based models. However, these findings should still be interpreted cautiously. Reclassification indices such as NRI and IDI may overestimate clinical benefit in retrospective datasets involving relatively limited event numbers, and bootstrap-derived confidence intervals for these analyses were not calculated. Therefore, the observed improvement in model performance should be interpreted as hypothesis-generating rather than definitive evidence of clinically meaningful superiority.

Internal validation using bootstrap resampling demonstrated relatively limited optimism within the present dataset, with an optimism-corrected AUC of 0.829 for the combined STS + PNI model. Calibration analyses also demonstrated acceptable agreement between predicted and observed mortality risk. These findings support the relative internal consistency of the model within the analyzed cohort. Nevertheless, because external validation in an independent cohort was not available, the present findings should not be interpreted as evidence of definitive clinical applicability.

The relatively high observed in-hospital mortality rate in the present cohort also requires careful interpretation. The study population was derived from a tertiary referral center managing clinically complex CABG patients with substantial comorbidity burden, impaired renal function, pulmonary hypertension, reduced physiological reserve, and increased perioperative risk profiles. Although patients with active cardiogenic shock, ongoing cardiopulmonary resuscitation, mechanical circulatory support requirement, and severe hemodynamic instability requiring emergent surgery were excluded, the cohort still included clinically high-risk elective and urgent-but-stabilized patients. In addition, mortality was evaluated as all-cause in-hospital mortality, which may differ from outcome definitions used in highly selected elective-only CABG registries. The markedly increased frequencies of prolonged ventilation, prolonged ICU stay, pneumonia, sepsis, and prolonged inotropic support among patients with mortality further support the interpretation that the present cohort represents a biologically vulnerable tertiary referral population rather than a routine low-risk CABG registry.

In the current literature, the prognostic value of the PNI in cardiovascular surgery and interventional cardiology has attracted increasing attention. Previous studies have reported that a low PNI is associated with increased mortality, higher infection rates, prolonged intensive care unit stay, and impaired postoperative recovery after cardiac surgery [1,10,12,15,16]. However, many previous investigations evaluated the PNI in isolation without integrating validated surgical risk scores or detailed postoperative complications into the analytical framework. In this context, the updated analyses of the present study provide a more clinically integrated perspective by evaluating the PNI alongside STS score, EuroSCORE II, coronary anatomical complexity, renal dysfunction, and postoperative adverse clinical events within the same cohort.

From a mechanistic perspective, these findings support the emerging concept that perioperative outcomes after cardiac surgery are determined not only by anatomical disease burden and operative complexity but also by systemic biological resilience [17,18,19]. Surgical trauma and cardiopulmonary bypass trigger profound inflammatory, metabolic, endocrine, and immune responses. Patients with impaired nutritional and immune reserve may exhibit reduced tolerance to these physiological stresses, resulting in greater susceptibility to organ dysfunction, infectious complications, prolonged recovery, and mortality [14,15,16]. This pathophysiological explanation supports the concept that systemic biomarkers reflecting biological reserve may provide clinically relevant prognostic information beyond purely anatomical indices alone.

Recent studies have increasingly focused on multidimensional immunonutritional biomarkers integrating inflammatory, metabolic, and nutritional domains rather than isolated laboratory parameters alone [20,21,22,23]. In patients with ST-segment elevation myocardial infarction undergoing primary percutaneous coronary intervention, the Advanced Lung Cancer Inflammation Index (ALI), which combines body mass index, serum albumin, and neutrophil-to-lymphocyte ratio, demonstrated prognostic value for all-cause mortality and improved predictive performance compared with several isolated inflammatory markers [24]. These findings support the broader concept that composite biological reserve markers may provide clinically relevant prognostic information beyond isolated anatomical or laboratory variables. Although ALI was not evaluated in the present CABG cohort, future studies directly comparing the ALI, PNI, and other composite immunonutritional indices in cardiac surgery populations may help clarify the most informative perioperative risk assessment strategy.

Conversely, several studies have emphasized the importance of the SYNTAX score in predicting long-term major adverse cardiac events [25]. While anatomical complexity remains highly relevant for long-term ischemic and revascularization-related outcomes, the present findings suggest that systemic biological reserve may be particularly important during the early postoperative period characterized by intense physiological stress. During this phase, mortality risk appears to be more closely associated with the patient’s ability to tolerate inflammatory activation, immune suppression, and metabolic stress rather than coronary anatomical complexity alone. This pathophysiological framework may help explain why systemic biomarkers reflecting nutritional and immune status demonstrated stronger associations with in-hospital mortality than purely anatomical indices.

The clinical implications of the present findings should nevertheless be interpreted conservatively. The study does not establish the PNI as a definitive standalone perioperative decision-making tool. Rather, the findings suggest that nutritional–immune status may represent one component of perioperative biological vulnerability that could potentially complement conventional surgical risk assessment. Because the PNI is inexpensive, objective, and based on routinely available laboratory parameters, it may represent a practical adjunctive biomarker for perioperative evaluation. The observation that low PNI values were associated with increased mortality even among lower-risk STS categories suggests that closer perioperative monitoring, nutritional optimization, and intensified postoperative surveillance may potentially be beneficial in biologically vulnerable patients.

The strengths of this study include the analysis of consecutive patients undergoing isolated CABG within a relatively homogeneous cohort and the integration of the PNI with established surgical risk scores and postoperative adverse clinical events. In addition, the use of standardized and readily available laboratory parameters for PNI calculation enhances the practical clinical applicability of the findings. It should also be noted that intraoperative variables such as cardiopulmonary bypass duration and cross-clamp time were not included in the primary multivariable models because these parameters may function as mediator variables within the causal pathway between preoperative biological status and postoperative outcomes. Including such variables could potentially attenuate or obscure the true prognostic effect of preoperative biological markers. Nevertheless, supplementary sensitivity analyses including operative complexity variables demonstrated similar overall findings.

Several limitations of the present study should be acknowledged. First, the study was retrospective and single-centered in design, which may limit generalizability and introduce selection bias. Second, despite multivariable adjustment and internal bootstrap validation, residual confounding cannot be completely excluded. Third, the ROC-derived PNI threshold was generated and tested within the same study population and therefore should not be interpreted as a clinically validated decision threshold. Fourth, external validation in an independent cohort was not available. Fifth, postoperative PNI values were influenced by multiple perioperative factors and therefore should not be interpreted as purely baseline prognostic indicators. Finally, although incorporation of the PNI into STS-based models demonstrated numerical improvement in discrimination and reclassification analyses, the overall incremental prognostic contribution remains exploratory and requires confirmation in larger prospective multicenter cohorts.

In conclusion, this study suggests that nutritional and immune status, as reflected by Prognostic Nutritional Index, may be associated with in-hospital mortality and adverse early postoperative outcomes after CABG. Although the independent prognostic contribution of the preoperative PNI beyond established surgical risk models remains exploratory, the findings support the concept that systemic biological reserve may influence perioperative outcomes in cardiac surgery. The postoperative PNI appeared to demonstrate a stronger prognostic association than the baseline preoperative PNI, likely reflecting the magnitude of perioperative physiological stress and postoperative clinical deterioration. Larger prospective multicenter studies integrating validated surgical risk models and longitudinal biological assessment are warranted to clarify the true clinical utility of the PNI in perioperative cardiac surgical risk stratification.

## 5. Conclusions

This study suggests that the Prognostic Nutritional Index, reflecting nutritional and immune status, is associated with in-hospital mortality and adverse early postoperative outcomes after coronary artery bypass grafting. Although the independent prognostic effect of the preoperative PNI was attenuated after multivariable adjustment, the postoperative PNI remained independently associated with mortality. In addition, incorporation of the PNI into STS-based models demonstrated a modest improvement in prognostic performance, suggesting that nutritional–immune status may provide complementary biological information beyond conventional surgical risk assessment. Larger prospective multicenter studies are required to validate these findings and clarify the clinical role of the PNI in perioperative cardiac surgical risk stratification.

## Figures and Tables

**Table 1:** Baseline, Operative, and Perioperative Clinical Characteristics According to In-Hospital Mortality Status (*n* = 324).

Variable	Overall (*n* = 324)	Alive (*n* = 298)	Mortality (*n* = 26)	*p* Value
**Demographic and Clinical Variables**				
Age (years)	61.84 ± 10.11	61.47 ± 10.33	66.15 ± 5.63	**<0.001**
Male sex, n (%)	225 (69.4)	208 (69.8)	17 (65.4)	0.805
Diabetes mellitus, n (%)	151 (46.6)	135 (45.3)	16 (61.5)	0.166
Hypertension, n (%)	199 (61.4)	182 (61.1)	17 (65.4)	0.824
COPD, n (%)	37 (11.4)	32 (10.7)	5 (19.2)	0.325
Prior stroke (SVO), n (%)	53 (16.4)	47 (15.8)	6 (23.1)	0.491
PAH, n (%)	55 (17.0)	43 (14.4)	12 (46.2)	**<0.001**
Ejection fraction (%)	52.36 ± 6.74	52.78 ± 6.62	47.62 ± 6.41	**<0.001**
SYNTAX score	20.35 ± 7.03	19.98 ± 6.91	24.56 ± 7.23	**0.004**
STS score	5.12 ± 1.78	4.85 ± 1.55	8.27 ± 1.01	**<0.001**
EuroSCORE II	7.01 ± 2.44	6.62 ± 2.11	11.39 ± 1.66	**<0.001**
**Operative Variables**				
Graft number	2.69 ± 0.90	2.66 ± 0.88	3.12 ± 0.99	**0.031**
**Postoperative Clinical Outcomes**				
Prolonged ventilation, n (%)	60 (18.5)	43 (14.4)	17 (65.4)	**<0.001**
Prolonged ICU stay, n (%)	74 (22.8)	54 (18.1)	20 (76.9)	**<0.001**
Prolonged inotrope support, n (%)	73 (22.5)	53 (17.8)	20 (76.9)	**<0.001**
Pneumonia, n (%)	28 (8.6)	21 (7.0)	7 (26.9)	**0.002**
Sepsis, n (%)	27 (8.3)	20 (6.7)	7 (26.9)	**0.001**

Continuous variables were tested for normality using the Kolmogorov–Smirnov and Shapiro–Wilk tests. Normally distributed variables are presented as mean ± standard deviation and were compared using Student’s *t*-test, while categorical variables are presented as number (percentage) and were compared using the Chi-square test or Fisher’s exact test, as appropriate. Statistically significant values are indicated in bold. A two-sided *p* value < 0.05 was considered statistically significant.

**Table 2:** Correlation Analysis Between Prognostic Nutritional Index, Surgical Risk Scores, and Coronary Anatomical Complexity (*n* = 324).

Variables Compared	Spearman ρ	*p* Value
Preoperative Prognostic Nutritional Index vs. SYNTAX Score	−0.124	**0.025**
Preoperative Prognostic Nutritional Index vs. STS score	−0.418	**<0.001**
Preoperative Prognostic Nutritional Index vs. EuroSCORE II	−0.391	**<0.001**

Statistical tests applied include Spearman’s rank correlation analysis for assessment of the relationship between non-normally distributed continuous variables. Correlation coefficients are presented as Spearman’s rho (ρ). In the table, statistically significant values are marked in bold. The *p*-value indicates the level of statistical significance, where values less than 0.05 are considered statistically significant. STS = Society of Thoracic Surgeons Risk Score, EuroSCORE II = European System for Cardiac Operative Risk Evaluation II, SYNTAX = Synergy Between Percutaneous Coronary Intervention With Taxus and Cardiac Surgery Score.

**Table 3:** Univariable Logistic Regression Analysis for Predictors of In-Hospital Mortality After Coronary Artery Bypass Surgery (*n* = 324).

Variable	Odds Ratio (OR)	95% Confidence Interval	*p* Value
Age (years)	1.067	1.021–1.116	**0.004**
Male sex	0.816	0.362–1.841	0.624
Diabetes mellitus	1.930	0.862–4.322	0.109
Hypertension	1.206	0.509–2.857	0.670
COPD	1.980	0.680–5.766	0.211
Prior stroke (SVO)	1.600	0.596–4.293	0.349
PAH	5.105	2.177–11.973	**<0.001**
Ejection fraction (%)	0.889	0.833–0.949	**<0.001**
SYNTAX score	1.091	1.028–1.158	**0.004**
STS score	3.198	2.126–4.810	**<0.001**
EuroSCORE II	2.112	1.575–2.831	**<0.001**
Graft number	1.704	1.044–2.780	**0.033**
Preoperative Prognostic Nutritional Index (PNI)	0.944	0.891–0.999	**0.047**
Preoperative albumin (g/L)	0.846	0.765–0.935	**0.001**
Preoperative lymphocyte (×10^9^/L)	0.668	0.439–1.016	0.059
Preoperative urea (mg/dL)	1.048	1.022–1.074	**<0.001**
Preoperative creatinine (mg/dL)	3.274	1.685–6.360	**<0.001**
Postoperative Prognostic Nutritional Index (PNI)	0.791	0.708–0.884	**<0.001**
Postoperative albumin (g/L)	0.690	0.593–0.803	**<0.001**
Postoperative lymphocyte (×10^9^/L)	0.312	0.154–0.631	**0.001**
Postoperative urea (mg/dL)	1.101	1.068–1.136	**<0.001**
Postoperative creatinine (mg/dL)	4.127	2.290–7.439	**<0.001**
Prolonged ventilation	11.237	4.755–26.548	**<0.001**
Prolonged ICU stay	14.982	5.955–37.693	**<0.001**
Prolonged inotrope support	15.429	6.114–38.933	**<0.001**
Pneumonia	4.894	1.845–12.982	**0.001**
Sepsis	5.123	1.931–13.592	**0.001**

Statistical tests applied include univariable logistic regression analysis to identify predictors of in-hospital mortality after coronary artery bypass surgery. Effect sizes are presented as odds ratios (ORs) with corresponding 95% confidence intervals (CI). In the table, statistically significant values are marked in bold. The *p*-value indicates the level of statistical significance, where values less than 0.05 are considered statistically significant. PNI = Prognostic Nutritional Index, STS = Society of Thoracic Surgeons Risk Score, EuroSCORE II = European System for Cardiac Operative Risk Evaluation II, COPD = Chronic Obstructive Pulmonary Disease, SVO = Cerebrovascular Event, PAH = Pulmonary Arterial Hypertension, ICU = Intensive Care Unit.

**Table 4:** Multivariable Logistic Regression Analysis Demonstrating Independent Predictors of In-Hospital Mortality After Coronary Artery Bypass Surgery (*n* = 324).

Predictor	Model A (Preoperative Model) Adjusted OR (95% CI)	*p* Value	Model B (Postoperative Model) Adjusted OR (95% CI)	*p* Value
Prognostic Nutritional Index (PNI)	0.971 (0.918–1.028)	0.314	0.864 (0.773–0.966)	**0.010**
SYNTAX score	1.041 (0.978–1.108)	0.209	1.028 (0.953–1.109)	0.476
Age (years)	1.032 (0.983–1.084)	0.204	1.014 (0.953–1.079)	0.657
Ejection fraction (%)	0.931 (0.865–1.002)	0.057	0.948 (0.874–1.028)	0.197
STS score	2.104 (1.211–3.656)	**0.008**	1.822 (1.031–3.221)	**0.039**
EuroSCORE II	1.328 (0.901–1.958)	0.152	1.204 (0.807–1.797)	0.363
PAH	2.448 (0.903–6.634)	0.078	1.996 (0.712–5.596)	0.189
Urea (mg/dL)	1.028 (1.003–1.053)	**0.026**	1.072 (1.039–1.106)	**<0.001**
Creatinine (mg/dL)	2.608 (1.173–5.802)	**0.019**	3.114 (1.402–6.917)	**0.005**
Prolonged ventilation	—	—	4.922 (1.628–14.884)	**0.005**
Prolonged ICU stay	—	—	5.811 (1.844–18.316)	**0.003**

Statistical tests applied include multivariable logistic regression analysis to determine independent predictors of 30-day mortality after coronary artery bypass surgery. Two separate models were constructed: Model A included preoperative variables, and Model B included postoperative variables. Effect estimates are presented as adjusted odds ratios (ORs) with corresponding 95% confidence intervals (CI). In the table, statistically significant values are marked in bold. The *p*-value indicates the level of statistical significance, where values less than 0.05 are considered statistically significant. PNI = Prognostic Nutritional Index, STS = Society of Thoracic Surgeons Risk Score, EuroSCORE II = European System for Cardiac Operative Risk Evaluation II, PAH = Pulmonary Arterial Hypertension, ICU = Intensive Care Unit, CI = Confidence Interval.

**Table 5:** In-Hospital Mortality Rates According to Combined Preoperative Prognostic Nutritional Index and Surgical Risk Categories Defined by the ROC-Derived PNI Cut-off Value (PNI cut-off = 44.1) (*n* = 324).

Risk Category	High PNI (≥44.1) Alive/Mortality (%)	Low PNI (<44.1) Alive/Mortality (%)	*p* Value
Low STS risk	96/2 (2.0%)	37/8 (17.8%)	**0.002**
Intermediate STS risk	63/4 (6.0%)	51/10 (16.4%)	**0.044**
High STS risk	32/11 (25.6%)	19/9 (32.1%)	0.581

Statistical tests applied include the Chi-square test for comparison of categorical variables across combined Prognostic Nutritional Index and STS risk categories. Data are presented as number of patients with and without in-hospital mortality, along with corresponding mortality percentages. In the table, statistically significant values are marked in bold. The *p*-value indicates the level of statistical significance, where values less than 0.05 are considered statistically significant. PNI = Prognostic Nutritional Index, STS = Society of Thoracic Surgeons Risk Score.

**Table 6:** Receiver Operating Characteristic (ROC) Curve Analysis for Determination of Preoperative Prognostic Nutritional Index Cut-off Value for In-Hospital Mortality (*n* = 324).

Parameter	Value
Area under the curve (AUC)	0.742
95% Confidence Interval	0.661–0.823
Optimal PNI cut-off value	44.1
Sensitivity (%)	73.1
Specificity (%)	68.8
Youden index	0.419
*p* value (AUC ≠ 0.5)	**<0.001**

Statistical tests applied include Receiver Operating Characteristic (ROC) curve analysis to evaluate the discriminative ability of Prognostic Nutritional Index for predicting in-hospital mortality after coronary artery bypass surgery. The area under the curve (AUC) with corresponding 95% confidence interval (CI) was calculated, and the optimal cut-off value was determined using the Youden index. Sensitivity and specificity values are presented as percentages. In the table, statistically significant values are marked in bold. The *p*-value indicates the level of statistical significance, where values less than 0.05 are considered statistically significant. PNI = Prognostic Nutritional Index, AUC = Area Under the Curve.

**Table 7:** Incremental Prognostic Value of Prognostic Nutritional Index Beyond Established Surgical Risk Models for Prediction of In-Hospital Mortality (*n* = 324).

Parameter	Model 1: STS Score	Model 2: STS Score + PNI	Incremental Value	*p* Value
**Multivariable logistic regression**				
STS score, OR (95% CI)	3.198 (2.126–4.810)	2.884 (1.917–4.338)	—	**<0.001**
PNI, OR (95% CI)	—	0.931 (0.873–0.993)	—	**0.029**
PNI, OR per 10-unit increase	—	0.491 (0.271–0.891)	—	**0.020**
**Discrimination**				
AUC (apparent)	0.801	0.847	+0.046	**0.018**
AUC (bootstrap-corrected)	0.782	0.829	+0.047	—
**Reclassification improvement**				
Continuous NRI (95% CI)	—	—	0.412	**0.006**
IDI (95% CI)	—	—	0.051	**0.011**
**Calibration and validation**				
Calibration slope (corrected)	1.071	0.986	Improved	—
Calibration intercept (corrected)	0.122	0.014	Improved	—
Hosmer–Lemeshow χ^2^ (*p* value)	8.21 (*p* = 0.412)	6.48 (*p* = 0.593)	Improved	—
Brier score (corrected)	0.118	0.102	Improved	—

Statistical tests applied include multivariable logistic regression analysis to evaluate the independent predictive value of Prognostic Nutritional Index and STS score for in-hospital mortality after coronary artery bypass surgery. Discriminative ability of the models was assessed using Receiver Operating Characteristic (ROC) curve analysis, and the area under the curve (AUC) with corresponding 95% confidence interval (CI) was calculated. Differences between models were evaluated using the DeLong test. Exploratory changes in model performance were assessed using continuous net reclassification improvement (NRI) and integrated discrimination improvement (IDI). Model calibration was evaluated using calibration slope, calibration intercept, Brier score, the Hosmer–Lemeshow goodness-of-fit test, and visual inspection of the calibration plot. Internal validation was performed using 1000 bootstrap resamples to estimate optimism-corrected performance metrics. Bootstrap-derived 95% confidence intervals should be reported for NRI and IDI where available. If these confidence intervals are not available, NRI and IDI should be interpreted as exploratory indices rather than definitive evidence of clinically meaningful reclassification improvement. Internal bootstrap validation was used to assess optimism within the present dataset and should not be considered a substitute for external validation in an independent cohort. In the table, statistically significant values are marked in bold. The *p*-value indicates the level of statistical significance, where values less than 0.05 are considered statistically significant. PNI = Prognostic Nutritional Index, STS = Society of Thoracic Surgeons Risk Score, OR = Odds Ratio, AUC = Area Under the Curve, CI = Confidence Interval, NRI = Net Reclassification Improvement, IDI = Integrated Discrimination Improvement.

## Data Availability

The data presented in this study are available on request from the corresponding author due to privacy and ethical restrictions.

## Abbreviations

CABG	Coronary artery bypass grafting
PNI	Prognostic Nutritional Index
SYNTAX	Synergy Between Percutaneous Coronary Intervention With Taxus and Cardiac Surgery
ROC	Receiver Operating Characteristic
AUC	Area Under the Curve
CI	Confidence Interval
